# Characteristics of users and implications for the use of complementary and alternative medicine in Ghanaian cancer patients undergoing radiotherapy and chemotherapy: a cross- sectional study

**DOI:** 10.1186/1472-6882-13-16

**Published:** 2013-01-19

**Authors:** Joel Yarney, Andrew Donkor, Samuel Y Opoku, Lily Yarney, Isaac Agyeman-Duah, Alice C Abakah, Emmanuel Asampong

**Affiliations:** 1 National Centre for Radiotherapy and Nuclear Medicine, Korle Bu Teaching Hospital, Accra, Ghana; 2 School of Allied Health Sciences, University of Ghana, Accra, Ghana; 3 School of Public Health, University of Ghana, Accra, Ghana; 4 Department of Mathematics and Statistics, Accra, Ghana

**Keywords:** Complementary, Alternative, Medicine, Radiotherapy, Chemotherapy, Cancer, Traditional, User-characteristics, Toxicity

## Abstract

**Background:**

There is widespread use of Complementary and Alternative Medicine (CAM) in Ghana, driven by cultural consideration and paradigm to disease causation. Whether there is concurrent use of conventional medicine and CAM in cancer patients is unknown. This study investigates the prevalence, pattern and predictors of CAM use in cancer patients. Overlapping toxicity, sources of information, and whether users inform their doctor about CAM use is examined.

**Method:**

Cross-sectional study using a questionnaire administered to cancer patients, who were receiving radiotherapy and or chemotherapy or had recently completed treatment at a single institution was used.

**Results:**

Ninety eight patients participated in the study with a mean age of 55.5 (18–89), made up of 51% females. Married individuals formed 56% of the respondents, whilst 49% had either secondary or tertiary education. Head and neck cancer patients were 15.3%, breast (21.4%), abdomen/pelvic cancers constituted (52%).Seventy seven (78.6%) patients received radiotherapy only, 16.3% received radiation and chemotherapy and 5.3% had chemotherapy only.

Ninety five patients were diagnosed of cancer within the past 24 months,73.5% were CAM users as follows; massage(66.3%), herbal(59.2%), mega vitamins(55.1%), Chinese medicine(53.1%),and prayer(42.9%). Sixty eight percent were treated with curative intent. Overlapping toxicity was reported. Majority (83.3%) of users had not informed their doctor about CAM use.

On univariate analysis, female (p=0.004) and palliative patients, p=0.032 were more likely to be CAM users. Multivariate analysis identified female (p<0.01), as significant for use, whilst head and neck site was significant for non use (p<0.028). Young, married and highly educated individuals are more likely to use CAM.

Friends and Media are the main sources of information on CAM. There was increase in CAM use after the diagnosis of cancer mainly for Chinese Medicine and vitamins.

**Conclusion:**

There is high CAM usage among Cancer patients, comparable to use in the general population, there is concurrent use of CAM and conventional medicine with reported overlapping toxicity but without informing Oncologist about use. Women and palliative patients are more likely to use CAM. Doctor patient communication on herbal-radiotherapy and drug treatment interaction needs to be strengthened. Standardization and regulation of CAM use is paramount.

## Background

Complementary and alternative medicine (CAM) has been defined as diagnosis, treatment or prevention that complements mainstream medicine by contributing to a common whole, by satisfying a demand not met by orthodoxy or by diversifying the conceptual framework of medicine [1,2].

Elements of what is referred to as CAM in contemporary literature were main stream medicine in West Africa prior to the arrival of “Western Medicine”, it is therefore not a new phenomenon. In Ghana, about 70% of the population depend on traditional medicine for their health care. There is approximately one traditional medicine practitioner for every 400 people, compared to one allopathic doctor for every 12, 000 people [3].

Whether an individual uses CAM or not is related to social and cultural considerations, including paradigm to disease causation. Chronic disease including cancer is attributed to the following; poor diets, poor lifestyle practices, heredity, physical factors, the environment, spiritual factors and psychological factors [4], health seeking behaviour is informed by these factors. In current times there is often a conflict between legitimized orthodox medicine and delegitimized traditional herbs and remedies.

The cost of orthodox medicine is often prohibitive to many individuals, for a large proportion of individuals who cannot afford these medicines, CAM remains the only alternative. Additionally the recent proliferation of media that promote the use of all forms of CAM seem to influence its use. Unlike conventional therapies that are regulated by law, and require practitioners to be registered and regulated, CAM practioners are difficult to track, consequences of their practice including untoward effects of the treatments are not documented.

Cancer is the third leading cause of death in developing countries [5]. Survival has improved over the years for many cancers resulting from improvement in conventional therapies and early presentation [6,7]. Despite this improvement, the effects of the treatment may still be a major concern to patients sufficiently for them to consider non-conventional treatment [8]. Indeed, most patients in the developing world including Ghana are diagnosed at a stage when most conventional therapies fail; this leads to a vicious cycle in which patients present late on the understanding that nothing can be done anyway, therefore they may as well resort to other therapies [9].

Acute side effects of chemotherapy and radiotherapy can occasionally be life threatening, it may affect patient compliance in addition to making them vulnerable to the adoption of alternative forms of treatment which promise cure. There may be overlapping side effects of treatment, which worsen the side effects that are caused by conventional therapies with a potential to have a negative influence on compliance to treatment thereby diminishing outcomes for conventional therapy, an example is gastrointestinal toxicity due to shark cartilage, and used by many for treatment of cancer even though it has no anti-tumour activity in the laboratory [10,11]. There could be drug-herb- vitamin interaction that works to reduce the efficacy of chemotherapeutic and hormonal treatment. The use of antioxidants and vitamin C during radiation therapy and chemotherapy could have a negative impact on outcomes [12]. In spite of these interactions, majority of patients do not inform their physician about concurrent use.

Literature from Sub Saharan Africa with respect to the use of CAM in Cancer patients is sparse, moreover demography of patients that are likely to use CAM, and reasons for use may be different from what pertains in developed countries because it is related to culture. We do not know much about concurrent use of CAM, and its potential consequences.

We therefore undertook to determine the following; (i) prevalence of CAM use among cancer patients, reasons for use, whether it is used concurrently with conventional therapies, whether they inform their Oncologist about concurrent use, and possible overlapping toxicity,(ii)sources of information on CAM, (iii) demography (age, sex, marital status, and level of education of patients who use CAM,(iv) whether the use of CAM is influenced by the intention to treat (palliative versus curative),and disease site and (v) effect of CAM use on treatment compliance.

## Methods

A cross-sectional descriptive survey design was used to collect data employing a questionnaire Additional file 1. The questionnaire on the use of complementary and alternative medicine designed by Emmanuel Ezeome was adopted and modified [13]. It was based on the National Center for Complementary and Alternative Medicine in USA’s classification of CAM therapies into five categories. The questionnaire was used to study the use of complementary and alternative medicine by cancer patients in Nigeria. This questionnaire which has been validated was chosen because of the cultural and geographical proximity of the two countries.

The questionnaire was administered from 11th May 2010 to 25th June 2010 after pretesting among 5 patients randomly selected from the outpatient department of the National Center for Radiotherapy and Nuclear Medicine of Korle Bu Teaching Hospital, Accra, the largest hospital in Ghana. The respondents had previously received anti-cancer therapy or were either undergoing radiotherapy or chemotherapy or both, they may or may not have submitted to Surgery in the past. Based on their responses, appropriate changes were made to the questionnaire.

Consent was obtained from each participant prior to administration. The questionnaire was administered by one of the authors who read it out to illiterate patients, the rest completed it independently. Respondents were either waiting to be seen by a physician, or to be treated.

Respondents were selected by convenient sampling provided they meet the following criteria; they had to be 18 years of age and above, diagnosed of cancer and referred to the National Centre for Radiotherapy and Nuclear Medicine, Korle-Bu Teaching Hospital for management. Patients, who were blind, deaf and dumb, mentally retarded or being managed on emergency basis or with poor performance status were excluded, as were those who participated in the pilot study.

Clinical characteristics and intention to treat (palliative versus curative) were gleaned from the patient chart.

Ethical approval was obtained from the Ethical Review Committee of the School of Allied Health Sciences, College of Health Sciences, University of Ghana.

Confidentiality of the information that the patients provided was ensured.

The data collected was analysed using statistical package for social sciences (SPSS 16.0 software, Chicago, IL). Descriptive statistics of frequencies and percentages was used to describe the categorical variables as well as chi-square contingency table technique. Multivariate analysis was performed using binary logit regression software, SAS version 9.0 (Cary, NC 27513 USA). *P* - Value < 0.05 was considered significant.

## Results and discussion

### Patient characteristics

Ninety eight patients were interviewed, none of the patients approached to answer the questionnaire declined. Patient age ranged from 18 to 89, with a mean of 55.5 years and a standard deviation of 17.1. Male and female respondents were 48(49%) and 50 (51%) respectively, whilst 55 (56%) were married and 43 (44%) were either single or divorced. Fifty respondents representing 51% of patients had received basic education, whilst 48 of them representing 49% had undergone either secondary or tertiary education.

### Clinical characteristics

Fifteen respondents representing 15.3% had been diagnosed with cancer of the head and neck region, breast cancer constituted 21.4%, cancers of the abdomen/pelvic organs (mainly cervix, rectum, stomach and prostate) comprised 52%, and others; 11.3%. Five respondents (5.1%), received only chemotherapy, 16(16.3%), received both chemotherapy and radiotherapy, whilst 77 (78.6%) had radiotherapy only. 0ver 95% of patients were diagnosed of cancer within the past 24 months of the study. Sixty eight percent of respondents were treated with curative intent, whilst 22% were treated with palliative intent; the intention to treat was not available in 10% of cases examined.

Among CAM users, 62.7% noted not abandoning conventional treatment for CAM. Majority (83.3%) of the CAM users did not inform their doctors about the CAM they were using or had used in the past.

Reported side effects include gastric upsets, nausea, itching, headaches, diarrhoea, and stomach aches. Others are dizziness, unpleasant smell, water retention, loss of appetite, and bleeding.

### Pattern of CAM use

CAM was used by 73.5% of respondents whilst 26.5% had not used it at all. Table 1 describes the pattern of CAM use. Respondents used more than one CAM at a time. Excluding the column “hope to use” and “used in the past”, the top five CAM used are Massage (66.3%), Herbal (59.2%), Mega vitamins (55.1%), Chinese Medicine (53.1%) and Prayers (42.9%) in decreasing order of use.

**Table 1:** Pattern of CAM use

	**Frequency (n)**			
**Types of CAM used by patients**	**Used in the past**	**Used since this cancer**	**Hope to use**	**Both in the past and since this cancer**
**Biological Based Therapies**				
High dose/mega vitamins	3	41	2	13
Herbal	8	28	0	30
**Mind-Body Systems**				
Prayers	24	15	2	27
Rituals	1	30	3	1
music therapy	24	5	4	11
Relaxation	13	15	8	9
Support group	1	18	38	5
**Alternative Medical Systems**				
Chinese medicine	0	43	6	9
Indian medicine	0	13	16	1
Acupuncture	0	7	5	0
Homeopathy	0	9	8	0
**Manipulative and Body Based Therapies**				
Chiropractic	0	2	10	0
Osteopathy	2	8	6	1
Massage	3	24	1	41
Reflexology	1	1	11	2
**Energy Therapies**				
Electromagnetic	1	6	11	0
Therapeutic touch	0	3	10	0

### How patients got to know about CAM

When asked about how they got to know about the CAM they were using, they answered as follows; Friends (33.8%), Mass Media (24.6%), Family members (16.9%), Health personnel (9.6%), CAM Practitioners (6.2%), Church and Religious Groups (6.2%), and not sure (4.6%).

### Reasons for use or avoidance of CAM

Participants were asked to note their reasons for using CAM. Among the reasons given were; they wanted to just try anything (31.2%), based on faith and beliefs (21.9%), and believing the sickness was spiritual (15.6%). Other reasons given were, conventional treatment is too mechanical (12.5%), disappointed in conventional treatment (9.4%), and conventional treatment is too toxic (9.4%).

Benefits patients expected to derive from the CAM they used include; to fight the cancer (40.6%), to relieve symptoms of the cancer/conventional treatment (23.2%), and to relax or sleep (17.4%). Other expected benefits were to improve the emotional and physical well being (14.5%) and to assist in wound healing (4.3%).

Non- CAM users were asked to note why they did not use it. Some had no faith in the effectiveness of CAM, others never thought of CAM, or were discouraged by some users of CAM, and yet others believed in the conventional medicine they were receiving and thought CAM was unnecessary.

### Statistical analysis

Tables 2 and 3 depict details of univariate and logistic regression analysis respectively performed on the data derived from the questionnaire.

**Table 2:** Contingency table of Patient characteristics and CAM use

	**CAM**	**USE**		**chi square**
	**observed**	**expected**	**p-value**	**(X^2^)**
Male				
Yes	29	36.7		
No	43	35.3		
Sex			0.004	8.206
Female				
Yes	19	13.3		
No	7	12.7		
>50	27	25.7		
Yes				
No	45	46.3		
Age Range			0.539	0.385
≤50				
Yes	18	16.7		
No	8	9.3		
Married				
Yes	42	40.4		
No	30	31.6	0.463	0.561
Marital Status				
Single and Divorce				
Yes	13	14.6		
No	13	11.4		
Basic				
Yes	35	36.7		
No	37	35.3	0.427	0.63
Level of Education				
Secondary and Tertiary				
Yes	15	13.3		
No	11	12.7		
Head and Neck				
Yes	12	10		
No	17	14		
Breast				
Yes	38	34		
No	5	14	0.665	21.9
Cancer Cases	3	5		
Abdomino-Pelvic				
Yes				
No	4	7		
Other				
Yes	13	17		
No	16	7		
Curative	47	47.9		
Yes				
No	19	15.5		
Treatment Intent			0.032	6.9
Palliative				
Yes	21	20.1		
No	3	6.51		

**Table 3:** Results of logistic regression for the model

**Parameter**	**Df**	**Estimate (β)**	**Pr > chisq**	**Odds ratio**	**CI 95%**
Intercept	1	0.953	0.467		
Marital status	1	0.013	0.647	1.295	0.429- 3.912
Gender	1	−1.012	0.010	0.132	0.028 - 0.617
Age	1	−0.038	0.102	0.963	0.920 - 1.008
Education 0	1	0.544	0.256	0.982	0.189 – 5.104
1	1	−0.761	0.116	0.266	0.46 – 1.532
2	1	−0.345	0.549	0.404	0.059 – 2.768
Tumour site 0	1	−0.297	0.562	0.151	0.026 – 0.869
1	1	−1.476	0.028	0.046	0.005 – 0.421
2	1	0.177	0.811	0.242	0.026 - 2.263
Treatment Intent 0	1	0.199	0.686	0.730	0.102 – 5.208
1	1	−0.712	0.282	0.293	0.025 – 3.85

Marital status (married = 1, single=0) Gender(male=1, female=0); Age was used as a continuous variable Educ. (Basic = 0, Sec. = 1, Tert. = 2, uneduc. = 3) Tumour site (Abd/pel = 0, H/Neck = 1, Thorax = 2, other =3) Treatment Int. (curative = 0, Palliative = 1, unspecified =2).

Elements of what is referred to as CAM in contemporary literature are part of the lifestyle of Ghanaians and West Africans, and have been practised since antiquity. This is very different from what is reported in the literature from Europe and North America. Whether there is concurrent use of CAM and modern medicine in cancer patients, and its consequences are not documented in our environment. In this study we sought to establish whether there is concurrent use of CAM and main stream medicine, prevalence of CAM use in cancer patients, the kinds of CAM used, the potential influence of CAM use on compliance to treatment, side effects of CAM, and predictors of CAM use in a setting where there is a changing paradigm to disease causation.

Participants were approached and invited to participate in the completion of the questionnaire instead of using mailed questionnaire, none of the patients invited to participate in the study declined (response rate of 100%) . In mailed questionnaire, non CAM users are likely not to respond, thus introducing bias, to that extent, our method is advantageous.

The study was carried out in Korle Bu Teaching Hospital which provides tertiary services and therefore has a good mix of rural and urban clientele, drawn from all over the country but with majority from the south.

While some studies included only a single disease site, quite a number also involved multiple disease sites like ours and were not restricted to a particular CAM [13-16].

We found that 73.5% of cancer patients used CAM for various reasons. This figure is close to the reported prevalence of CAM use in the general Ghanaian population of 70%, most patients are therefore CAM users even before diagnosis and do not stop the practice because they are undergoing anti cancer treatment. CAM is used both for prevention, maintenance and curative purpose [3]. For instance it is believed that most diseases are caused by accumulation of mucus in the body, any treatment or concoction that removes mucus like laxatives is considered to have medicinal potential according to CAM practitioners. It is not uncommon to hear advocates of CAM use advertising this feature even to healthy individuals and encouraging its use to promote and maintain health. A study reported no difference in CAM use between cancer patients and non cancer patients, this is however not consistent with other studies [14,16].

In a review of 26 surveys from 13 countries carried out from 1977 to 1998, prevalence of CAM use in cancer patients was 31.4% with a range of 7% to 64% [17], more recent studies have reported 70.2% and 83.3% [15,18]. This may be a reflection of the instrument used, the definition of CAM and the sample size, nevertheless our reported figure falls within this range, the only difference is that in our setting, cancer patients have not resorted to its use only because of the diagnosis.

The most frequently used CAM types reported is a reflection of the perception of disease causation. A recent study on the causes of chronic disease including cancer in Ghana reported seven causes as follows; poor diets, poor lifestyle practices, heredity, physical factors, the environment, spiritual factors and psychological factors. In view of this, within an individual, there is often a conflict between the legitimized orthodox medicine and the often frowned upon local medicine since these ideas are well ingrained [4]. It is therefore not surprising that patients continue to use CAM in addition to orthodox medicine.

Massage, herbalism, vitamins, chinese medicine and prayers are the most subscribed CAM. This is not too different from studies carried out elsewhere, except that Massage and Spiritual Practice(Prayers) ranked high, compared to other cultural settings, but this is similar to the picture from Nigeria probably because of similarities in culture [13], the only deviation with respect to other reports in Africa is the high usage of Chinese Medicine in Ghana. Chinese medicine, Vitamins and Rituals witnessed the greatest increase in usage after the diagnosis of cancer. The least used CAM like osteopathy, reflexology and electromagnetic touch, may be a reflection of availability rather than preference for the others [16].

In Ghana, most conventional therapies are out of pocket payments similar to CAM, insurance cover or affordability may therefore not necessarily be a predictive factor. There is however a wide range in the cost of most CAM, ranging from very expensive to free. Resort to CAM use may be the only choice available when the cost of conventional therapy is high relative to the choice of a particular CAM that is available to the patient.

Some patients used CAM because they wanted to just try anything (31.2%); this is a reflection of doubt in the efficacy of conventional treatment. Majority of patients in the developing world including Ghana are diagnosed at a stage when most conventional therapies fail; this is a result of the absence of screening and educational programmes, as well as paradigm to disease causation, others also recur after varying periods of remission and majority become incurable with conventional therapies. In a study among newly diagnosed breast cancer patients, the causes of delayed presentation were: previous medical consultations 26(29.4%), ignorance 19(28.8%), fear of mastectomy 16(24.2%), herbal treatment 13(19.7%), prayer/prayer camps 13(19.7%) and financial incapability 12(18.2%). Fear of mastectomy 20(57.1%), herbal treatment 13(37.1%), financial incapability 11(31.4%) and prayers/prayer camps 10(28.6%) which were prominent causes of late presentation, were the main reasons for absconding [9]. A vicious cycle of late presentation emanating from paradigm to disease causation leading to diminished chances of cure and therefore reduced belief in the efficacy of conventional therapies ensues and increases subscription to CAM.

Whilst 40.6% of CAM users actually believed that CAM fights the cancer, majority used it to improve their quality of life by relieving symptoms caused by the cancer or conventional therapy(23.2%), relax or sleep(17.4%), for improvement in the emotional and physical well being (14.5%) and wound healing(4.3%). This finding is similar to that found in United States where patients resorted to CAM to improve their quality of life, boost the immune system and relieve symptoms [15] but is at variance with what is reported by patients from Nigeria, United Kingdom and Turkey where users expected CAM to directly cure their disease [13,19,20].

Positive results have been reported for some forms of CAM, for instance, acupuncture has been shown to relieve chemotherapy induced nausea and vomiting [21], self-hypnosis, massage or acupuncture induce pain relief [22], short term improvement in psychological well being with aromatherapy massage and relief of dyspnoea with acupuncture, acupressure or relaxation/breathing techniques [23].

Non users cited lack of belief in the efficacy of CAM, others were discouraged by previous users reflecting a negative experience with the use of CAM.

The reported side effects may be a result of the biological activity of the active ingredients in ingested CAM [24] or pesticide, fungal and bacterial contamination [25]. There may be use of incorrect plant species [26], absence of standardisation (leading to possible substitution, adulteration, incorrect dosing or preparation and inappropriate labelling or advertising) [27]. Some herbal preparations have toxic effects (kava causes hepatotoxicity), interact with prescription drugs (St John Wort), or cause surgical complications (garlic, ginko and ginseng may enhance bleeding and ginseng causes hypoglycaemia) [27,28].

Pelvic radiation causes inflammation of the bowel (enteritis and proctitis) in itself, additional gastrointestinal toxicity arising from any of the factors listed is likely to exacerbate diarrhoea and abdominal cramps.

In a study carried out in Ghana, 52.9% clients of traditional medicine and 75% orthodox medicine users claimed that the use of traditional medicine is not safe as compared to orthodox medicine, but only 17.1% and 5% of traditional medicines users and orthodox medicine users respectively described traditional medicine usage as remarkably safe and totally out of harm’s way [29].

The observation that 83.3% of CAM users had not informed their doctors about CAM use is troubling because of the issues associated with concurrent use as described including the negative effect of supplemental anti oxidant and vitamin administration during radiotherapy and chemotherapy [12] and the detrimental effect of vitamin E and Selinium as demonstrated in the SELECT study [30]. In a systematic review comprising 21 studies, 11% to 95% admitted CAM use, however between 20% and 77% of users did not disclose their CAM use to their Oncologist. Reasons cited include; doctors lack of enquiry, patients anticipation of doctors disapproval, disinterest, or inability to help, and patients perception that disclosure of CAM use is irrelevant to their conventional care [31].

Predictors of CAM use in our setting are gender and intention to treat on univariate analysis (table 2). Women and patients treated with palliative intent are more likely to use CAM, with p values of 0.004 and 0.032 respectively. Age, marital status, level of education and tumour site were not statistically significant. Our finding in relation to intention to treat is in agreement with those of Molassiotis et al., who reported greater use in patients treated with palliative intent or patients treated for cancers with poorer prognosis [16]. Kritoffersen et al. also reported higher prevalence of CAM use among cancer patients with poorer prognosis, other studies have reported otherwise [32].

Even in an environment of high CAM usage by the general population, gender continued to be significant on multivariate analysis (p=0.01) (table 3). Tumour site was also seen to be a significant factor affecting the use of CAM with a p-value of 0.028. It can be said that, those married are 29.5% more likely to use CAM than singles. Males are 86.8% less likely to use CAM than females. Similarly, a year increase in age reduces the use of CAM by 3.7%. In other words younger people have a higher tendency to use CAM. At the educational level, those with basic, secondary and tertiary education are 1.8%, 73.4% and 59.6% less likely to use CAM than the uneducated. As one moves from secondary to tertiary level of education, there is an increase in CAM usage but those with basic education have a higher usage of CAM. Tumours at the abdominal/pelvic, head and neck and thoracic areas are 84.9%, 95.4% and 75.8% respectively less likely than tumours at other parts of the body to use CAM, particularly head and neck patients are less likely to use CAM, this is probably due to the difficulty such patients have with swallowing. These findings are in agreement with most studies [13-16].

Media and friends constitute the greatest source of information on CAM. In spite of the fact that radio coverage in Ghana is almost hundred percent, its content with respect to traditional medicine is hardly regulated, with vendors making unsubstantiated claims that potentially influences patient behaviour and preference. The preparations on the market usually lack labels, and do not report side effects, dosing is usually verbal. In a recent publication, buyers were admonished to beware of the potential for ingested CAM to cause side effects or hinder the efficacy of conventional therapies [33]. Even though there is an attempt at regulation by the Traditional Medical Council, it does not go far enough. The Mampong Center for Research into Plant Medicine seeks to apply scientific protocols to the practice of traditional medicine.

Our study is limited by the rather low number of participants, non-probability sampling method used, and the fact that it was performed in a single institution. Additionally it is hospital based, there may be cancer patients who are CAM users but do not attend hospital at all, their responses especially in respect of reasons for subscription to CAM will be interesting. Nevertheless, it provides valuable insight into the behaviour of cancer patients with respect to CAM use in an environment where there is high patronage for CAM in the general population.

## Conclusion

There is high usage of CAM in Ghanaian cancer patients, and majority subscribe to CAM use concurrently with conventional medicine. The main reason for continued use is to improve quality of life including psychological boost. These patients are willing to share this information with their Oncologist, but health personnel fail to ask patients direct questions on CAM. Such patients tend to be female and are often treated with palliative intent. Overlapping toxicity between biological agents and radiotherapy and chemotherapy were reported, as were intake of agents that have the potential to reduce the efficacy of conventional therapies.

It is therefore recommended that Physicians should routinely ask patients about concurrent use of CAM and conventional therapies in order to provide appropriate advice. CAM practioners and advertisement in the media on CAM should be regulated. Public education on risk factors for cancer should be intensified in order to influence perceived causes of cancer and therefore behaviour. Psychological and Spiritual support should be added to patient care in order to ensure that evidence based information is provided to patients. A future study on health professionals’ attitude to CAM as well as a more detailed study of particular CAM patients use will be helpful in patient care.

## Competing interest

The authors have no competing interest including financial to report.

## Authors’ contribution

JY: Concept formulation and drafting of script. AD: Concept formulation, questionnaire administration and drafting of script. SO; Concept formulation and drafting of script. LY: Drafting of script. IA-D: Statistical analysis and drafting of script. AA: Statistical analysis and drafting of script. EA: Drafting of script. All authors read and approved the final manuscript.

## Pre-publication history

The pre-publication history for this paper can be accessed here:

http://www.biomedcentral.com/1472-6882/13/16/prepub

## Supplementary Material

Additional file 1 Questionnaire on CAM use in cancer patients undergoing Radiotherapy and Chemotherapy in Ghana. Click here for file
