# Supplementary material for: Characteristics of users and implications for the use of complementary and alternative medicine in Ghanaian cancer patients undergoing radiotherapy and chemotherapy: a cross- sectional study
Source: BMC Complement Altern Med. 2013 Jan 19;13:16. doi: 10.1186/1472-6882-13-16 (PMC3565863; doi:10.1186/1472-6882-13-16)
Supplement: Additional file 1 — Questionnaire on CAM use in cancer patients undergoing Radiotherapy and Chemotherapy in Ghana. [file 1472-6882-13-16-S1.pdf]

APPENDIX I

INFORMED CONSENT FORM

NAME OF RESEARCHER: MR ANDREW DONKOR

P.O.BOX 913, OBUASI-GHANA

TEL.0245723364/0274067735

NAME OF INSTITUTION: SCHOOL OF ALLIED HEALTH SCIENCES

COLLEGE OF HEALTH SCIENCES

UNIVERSTIY OF GHANA

LEGON-ACCRA

PROJECT TITTLE: CROSS-SECTIONAL STUDY ON THE USE OF COMPLEMENTARY AND ALTERNATIVE TREATMENT MODALITIES BY CANCER PATIENTS IN GHANA.

I have been invited to take part in this study for the research titled above. My role in this study in this study is to complete an attached questionnaire.

I acknowledge that the research procedures have been explained to me and that any questions that I have asked have been explained to my satisfaction.

I have been informed of the alternatives to participation in this study including the right to not participate. I also understand that I may not benefit directly from the research and that my participation is totally voluntary.

I have also been informed that the confidentiality of the information I will provide will be safeguarded and that my privacy and anonymity will be ensured in the collection, storage and publication of the research material.

i…………………………….., have fully understood the aims, methods and conditions to participate in this study, I therefore consent to my participation.

…………………………………

Participant’s signature/thumbprint　　　　　　　　　Date

…………………………………

Researcher’s signature/thumbprint　　　　　　　　　Date

Supervisors:

1. **Dr. S.Y. Opoku**
   Radiotherapist (PhD)
   Radiotherapy Department, KBTH
   Accra, Ghana.
   Tel. 021676222

2. **Dr. Joel Yarney**
   Radiation Oncologist
   Radiotherapy Department, KBTH
   Accra, Ghana.
   Tel. 021676222

APPENDIX II

QUESTIONNAIRE ON THE USE OF COMPLEMENTARY AND ALTERNATIVE MEDICINE BY CANCER PATIENTS IN NIGERIA

This questionnaire is designed to find out to what percentage of cancer patients in our Ghana uses complementary and alternative medicine(s) as part of treatment of their condition. The responses you provide in no way affect your treatment. The findings of this research will help us to improve on our care for cancer patients. You may choose not to respond to any of the questions you are not comfortable with. We guarantee your confidentiality.

SECTION 'A' (DEMOGRAPHIC DATA)

1. Age: -------------- 2. Sex: Male-------- Female---------

3. Marital status: (A) Married (B) Not married (C) Widow (D) Divorced/Separated

4. Highest level of education attained: (A) Non (B) Primary (C) Post primary/Secondary (D) University/Polytechnic/College of education

5. Profession/Occupation: --

6. Level of Income per month:

(A) Less than GhC60.00/month (B) Less than GhC200.00/month

(C) More than GhC300.00/month

7. Religion: (A) Traditional religion (B) Christian (C) Muslim (D) Others (specify) –

SECTION 'B' (CONVENTIONAL CANCER TREATMENT)

8. Type of cancer? -- (Pls confirm from case note)

9. Stage of cancer? -- (Pls confirm from case note)

10. Treatment intent? ………………………………….(Pls confirm from case note)

11. How long have you had this disease? ---

12. How long have you been on treatment for this disease? ---------------------------------

13. What types of treatment have you received in the past for this cancer?

(A) Chemotherapy

(B) Surgery (specify)

(C) Radiotherapy

(D) Others (specify)

14. What type of treatment are you currently receiving for this disease? (A) Chemotherapy (B) Surgery (specify) (C) Radiotherapy (D) Others (specify) ---------

SECTION 'C' (COMPLEMENTARY AND ALTERNATIVE MEDICNE (CAM) USE)

15. Have you used any thing other than that given to you by a medical doctor to treat this cancer? (A) Yes (B) No, why? ....

If NO, don't answer the following questions. (Thank You).

16. If YES, below is a list of CAM people have used to treat cancer. I will lke to know which ones you have used in the past, which ones you have used since this cancer started and which ones you think you may used in future. You should mark all that applies to you for each CAM.

BIOLOGICAL BASED THERAPIES:

	**Used In the Past**	**Used Since This Cancer**	**Hope to Use**
High dose/ mega vitamins	………………	………………………	…………..
Herbal	………………	………………………	……………

Please give the name of the herbal clinic and medicine

MIND-BODY SYSTEMS

	**Used In the Past**	**Used Since This Cancer**	**Hope to Use**
Prayers	………………	………………………	…………..
Ritual sacrifices	………………	………………………	…………..
Music therapy	………………	………………………	…………..
Relaxation (hypnosis)	………………	………………………	…………..
Support group	………………	………………………	…………..

ALTERNATIVE MEDICAL SYSTEMS

	**Used In the Past**	**Used Since This Cancer**	**Hope to Use**
Chinese Medicine	………………	………………………	…………..
Indian Medicine	………………	………………………	…………..
Acupuncture	………………	………………………	…………..
Homeopathy	………………	………………………	…………..

MANIPULATIVE AND BODY BASED THERAPIES

	**Used In the Past**	**Used Since This Cancer**	**Hope to Use**
Chiropractic	………………	………………………	…………..
Osteopathy/ Bone setters	………………	………………………	…………..
Massage	………………	………………………	…………..
Reflexology	………………	………………………	…………..

ENERGY THERAPIES

	**Used In the Past**	**Used Since This Cancer**	**Hope to Use**
Bioelectromagnetics	………………	………………………	…………..
Therapeutic touch	………………	………………………	…………..

17. Are there other types of CAM not listed above which you have used?

(A) Yes (specify) -- (B) No-----

18. If you have used any CAM in this cancer, for how long have you used it? -------------

19. How frequently have you been using CAM in this cancer?

(A) Daily (B) weekly (C) Occasionally (D) Only once

20. How frequently have you visited a CAM practitioner since you were diagnosed to have cancer?

(A) Non (B) Once (C) Several times

21. What are your reasons for deciding to use CAM? ……………………………………………..

22. What benefits were you hoping to get from the CAM you used in this cancer?

....

23. Have you obtained any particular benefit from the CAM you used? (A) Yes (B) No

24. Did you experience any unwanted effect from the CAM you used in this cancer?

(A) Yes (specify) --- (B) No

25. How satisfied are you with the performance of the CAM you used in this cancer?

(A) Very satisfied (B) Satisfied (C) Disappointed

26 Have abandon conventional treatment tor CAM since you started CAM? (A) Yes (B) No

27. What were your reasons for abandoning conventional treatment for CAM since you started CAM? ---

28. Did you mention to the doctor in charge of this your cancer management that you have used/are using CAM? (A) Yes (B) No

29. If your doctor is not aware that you have used/are using CAM, what is it that makes you feel unwilling to discuss it with him/her?---
----

30. How did you come to know of the CAM you are using/have used? ....

31. How do you get your supply of CAM? ………………………………………………………………

32. How much do you estimate that you have spent on CAM in the last one year? -----------------------

33. What particular aspects of your CAM would you have wished is available in conventional medicine? ……………………………………………………………………………………..

34. Does any body supervise/guide you in the CAM you use?

(A) Yes (B) No

THANK YOU.
